# Glomerular filtration rate in patients with renal space-occupying masses using combination of SPECT ^99m^Tc-DTPA renal dynamic imaging and contrast-enhanced CT

**DOI:** 10.3389/fonc.2025.1737442

**Published:** 2026-01-12

**Authors:** Mingsheng Jia, Ling Jiang, Xiujuan Li, Shengdong Nie, Baojian Wang

**Affiliations:** 1 Department of Nuclear Medicine, The Second Affiliated Hospital of Shandong First Medical University, Taian, Shandong, China; 2 Department of Medical Equipment, The Second Affiliated Hospital of Shandong First Medical University and Shandong Academy of Medical Sciences, Taian, Shandong, China; 3 Medical Imaging Center, The Affiliated Taian City Central Hospital of Qingdao University, Taian, Shandong, China; 4 School of Health Science and Engineering, University of Shanghai for Science and Technology, Shanghai, China

**Keywords:** ^99m^Tc-DTPA, contrast-enhanced CT, glomerular filtration rate (GFR), renal dynamic imaging, SPECT

## Abstract

**Objective:**

To investigate changes in Glomerular Filtration Rate (GFR) and influencing factors in patients with renal space-occupying masses using a combination of single-photon emission computed tomography (SPECT) Tc-99m-labeled diethylenetriaminepentacetic acid (^99m^Tc-DTPA) renal dynamic imaging and contrast-enhanced computed tomography (CT).

**Methods:**

Fifty patients with renal masses underwent SPECT ^99m^Tc-DTPA renal dynamic imaging and contrast-enhanced CT scans, and the GFR of the diseased kidney was compared with that of the contralateral healthy kidney in the same patient. The correlation between the size of the masses and the reduction in GFR was analyzed. Besides, the relationships between the net increment CT values in the renal parenchymal phase and excretory phase with the GFR changes were analyzed.

**Results:**

The GFR of diseased kidneys was significantly lower than that of the healthy kidneys (*p* < 0.05). There was no correlation between the size of the masses and the reduction in GFR (r=0.23, *p*>0.05). A negative correlation was found between the net increment CT value in the parenchymal phase and the decline in GFR (r=-0.38, *p* < 0.05), while there was no correlation between the net increment CT value in the excretory phase and the decline in GFR (r=-0.26, *p*>0.05).

**Conclusion:**

The blood supply characteristics observed in contrast-enhanced CT scans of renal masses can influence the calculation of GFR in radionuclide renal dynamic imaging. These characteristics should be taken into account when interpreting GFR results.

## 1 Introduction

Glomerular filtration rate (GFR) is defined as the volume of fluid filtered from the glomerular capillaries into Bowman’s capsule per time unit. GFR is widely recognized as a critical index for the clinical evaluation of renal function (1–3). Single-photon emission computed tomography (SPECT) Tc-99m-labeled diethylenetriaminepentacetic acid (^99m^Tc-DTPA) renal dynamic imaging, utilizing the Gates method, is a technique employed to determine GFR. This method not only provides a dynamic visualization of renal processes (from blood perfusion and cortical uptake to excretion) but also enables the assessment of renal function and the calculation of GFR values (4, 5).

Accurate preoperative split renal GFR is crucial for surgical planning, particularly in nephron-sparing surgery. While SPECT ^99m^Tc-DTPA imaging is a standard for this purpose, its accuracy in the setting of renal masses remains underexplored. The Gates method assumes that renal ROI counts primarily originate from functioning nephrons. However, hypervascular renal tumors may sequester the tracer, potentially introducing ‘spill-in’ counts that do not reflect glomerular filtration, thereby confounding GFR calculation. Contrast-enhanced computed tomography (CT), routinely performed for tumor staging, provides detailed vascular mapping but is not quantitatively integrated into functional assessment.

Contrast-enhanced CT is routinely used for preoperative evaluation of renal space-occupying masses. This imaging modality not only allows for the assessment of the blood supply to the masses but also provides detailed visualization of the kidney itself and the surrounding perirenal anatomical structures.

Therefore, this study aimed to investigate the effects of renal masses on GFR by quantitatively integrating enhancement characteristics from contrast-enhanced CT with functional data from SPECT renal dynamic imaging, in order to provide a basis for more accurate renal function evaluation and to guide clinical decision-making.

## 2 Materials and methods

### 2.1 Patients

#### 2.1.1 Ethical statement

This retrospective study was conducted in accordance with the Declaration of Helsinki and was approved by the Institutional Review Board of the Second Affiliated Hospital of Shandong First Medical University (Approval No: R202103030075). The requirement for individual informed consent was waived due to the retrospective nature of the study.

The data of patients admitted to the Second Affiliated Hospital of Shandong First Medical University for renal space-occupying masses between July 2020 and April 2022 were retrospectively analyzed, provided they met the following selection criteria: 1) undergoing surgical treatment or needle biopsy for a single renal mass with definitive pathological results; 2) completion of ^99m^Tc-DTPA renal dynamic imaging with GFR assessment and contrast-enhanced renal CT within one week prior to surgery; 3) absence of acute or chronic conditions such as hypertension or diabetes that could potentially influence GFR, with renal blood biochemical indices within normal ranges; 4) normal GFR and imaging findings in the contralateral healthy kidney; and 5) compliance with medical ethics standards, including signed informed consent from all patients. Exclusion criteria included: 1) renal masses causing significant compression of the renal sinus or renal pelvis; 2) imaging quality insufficient for diagnostic purposes; 3) history of other renal diseases (e.g., glomerulonephritis, renal stones), heart failure, or use of nephrotoxic medications; and 4) presence of diabetes mellitus or uncontrolled hypertension, despite biochemical indices being within normal ranges, to minimize potential confounders on GFR.

A total of 50 patients who met the study criteria were enrolled, comprising 28 males and 22 females, with a mean age of 62.0 ± 12.2 years (range: 34–83 years). All patients presented with unilateral nephropathy, resulting in the inclusion of 50 kidneys, including left (n=23) and right (n=27) renal masses. Among the lesions, 45 were malignant tumors, and 5 were benign masses. The malignant tumors included clear cell carcinoma (n=40), papillary renal cell carcinoma (n=3), chromophobe cell carcinoma (n=1), and metastatic carcinoma (n=1). The benign lesions included renal angiomyolipoma (n=2), simple renal cyst (n=2), and renal eosinophilic adenoma (n=1) (**Table 1**). This retrospective study consecutively enrolled all eligible patients meeting the criteria within the specified period. The sample size, though limited, represents a homogeneous cohort for investigating the specific relationship between mass vascularity and SPECT-GFR.

**Table 1:** Patient characteristics.

Category	n	%
Number of patients (female/male)	50 (22/28)	
Age, years	62.0 (34–83)	
Number of masses (L/R)	50 (23/27)	
Diagnosis (Malignant)		
Clear cell carcinoma	40	80
Papillary renal cell carcinoma	3	6
Chromophobe cell carcinoma	1	2
Metastatic carcinoma	1	2
Total	45	90
Diagnosis (Benign)		
Renal angiomyolipoma	2	4
Simple renal cyst	2	4
Renal eosinophilic adenoma	1	2
Total	5	10

### 2.2 Renal dynamic imaging equipment and protocol

SPECT renal dynamic imaging combined with GFR measurement was performed using the Discovery NM/CT670 Pro dual-probe SPECT/CT system manufactured by GE, equipped with a low-energy, high-resolution parallel-hole collimator and a 16-row spiral CT. The imaging agent, technetium ^99m^Tc-DTPA, was supplied by Jinan Atomic High-tech Pharmaceutical Co., Ltd., with a radiochemical purity exceeding 95%. Prior to the examination, the patient consumed 300–500 ml of water 30 minutes beforehand and emptied their bladder. The patient was positioned supine on the examination table, with the probe placed in the lower body region. The imaging agent was administered as a bolus injection into the antecubital vein at a dose of 296 MBq (8 mCi), followed by a rapid flush with 10 ml of normal saline. The renal blood perfusion phase was immediately acquired at 2 seconds per frame for 30 frames (1 minute), followed by the renal function phase at 60 seconds per frame for 20 frames (20 minutes). The acquisition matrix was set to 64×64, with an energy peak of 140 keV. Radioactive counts for both full and empty syringes were recorded before and after the examination to ensure accurate dose administration. The administered dose was calculated from the counts of the full and empty syringes measured before and after injection using a dose calibrator. Kidney depth was automatically estimated and corrected for by the SPECT/CT system software as part of the Gates method GFR calculation.

### 2.3 Renal dynamic image processing and GFR analysis

**Figure 1:**
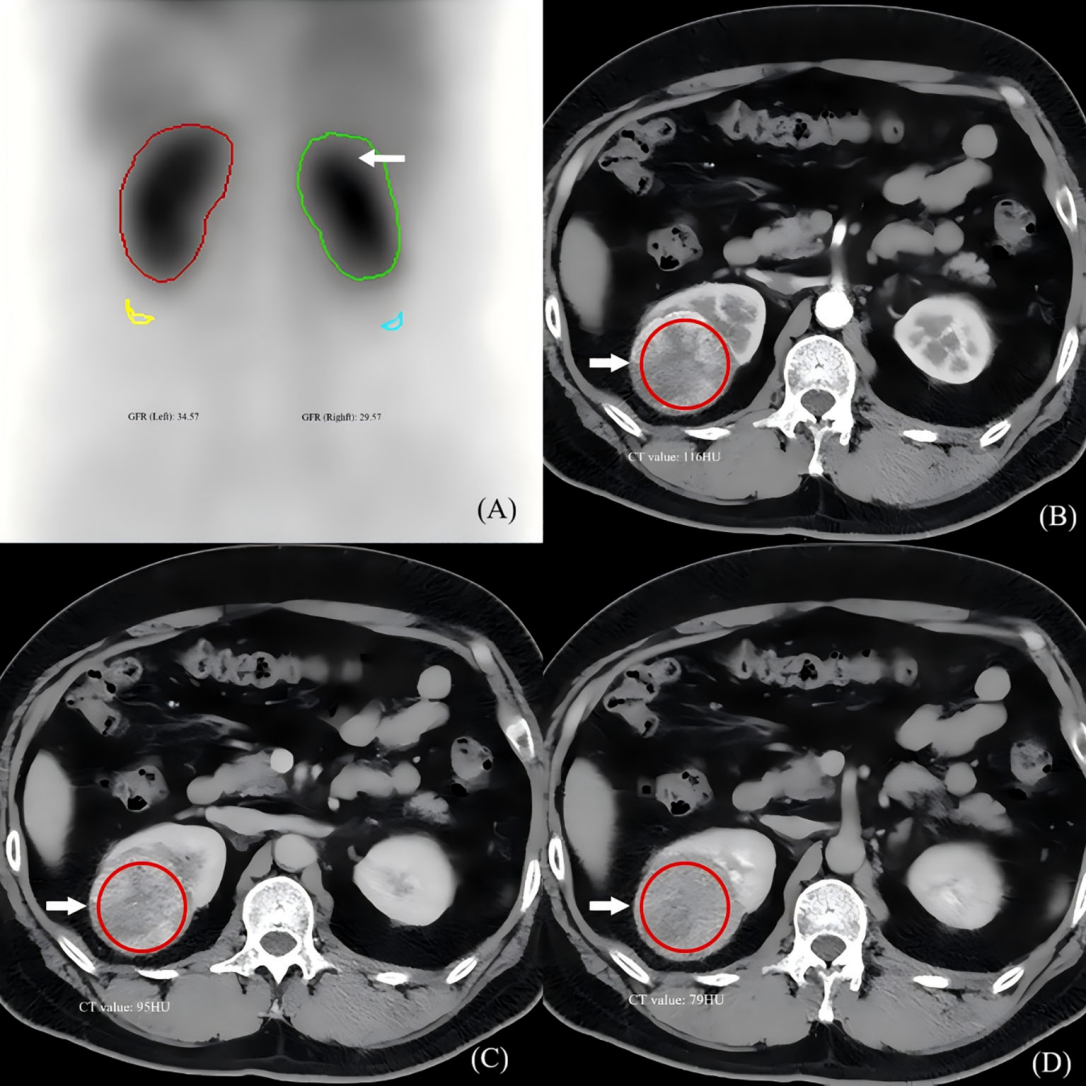
Multimodal imaging example of a renal mass influencing GFR calculation. **(A)** SPECT ^99m^Tc-DTPA renal function phase image (posterior view) in a 58-year-old female with a right upper pole mass (arrow). The regions of interest (ROIs) for the left (healthy) and right (diseased) kidneys are shown. The calculated GFR is lower on the diseased side. **(B–D)** Corresponding contrast-enhanced CT images at the same level: **(B)** corticomedullary phase, **(C)** nephrographic phase, and **(D)** excretory phase. The tumor (arrow) exhibits marked enhancement in the nephrographic phase. This vascular activity likely contributes to the renal ROI counts on SPECT, illustrating the potential mechanism for GFR overestimation in hypervascular masses.

Using the Renal Analysis function of the Xeleris Version 4.0 workstation, the patient’s height, weight, and other relevant information were input following the completion of the scan. The regions of interest (ROI) for both kidneys were carefully drawn to encompass the entire renal parenchyma while excluding the collecting system and the space-occupying mass itself, based on the co-registered CT images for anatomical guidance. The abdominal aorta ROI was placed on the early perfusion phase image. A semilunar background ROI was sketched below the outer edge of each kidney to correct for extra-renal activity. The computer program calculated the total GFR for both kidneys (**Figure 1**), as well as the individual GFR for each kidney, the time to peak (TP) for the left and right kidneys, and the half excretion time (T1/2) using the Gates method. Besides, the time-activity curve (TAC, nephrogram) for both kidneys was automatically generated. The separate GFR values were recorded as A (GFR for the healthy kidney) and B (GFR for the diseased kidney), with Y representing the percentage decrease in GFR for the diseased kidney, calculated as Y = (A-B)/A*100%.

### 2.4 CT scanning protocol and image analysis

Kidney routine plain scan and contrast-enhanced CT scan were performed using the Philips Brilliance iCT scanner. Patients were instructed to fast for 4–6 hours prior to the examination and were asked to drink approximately 300–500 ml of water within 30 minutes before the scan, without the need to hold urine. The CT scanning parameters were set as follows: 120 kV, 160 mAs, rotation speed of 0.5s/turn, pitch of 0.75, and a layer thickness of 5 mm. A non-ionic contrast agent, ioversol injection (350 mgI/ml), manufactured by Jiangsu Hengrui Pharmaceutical Co., Ltd., was administered intravenously at a dose of 80–100 ml through the antecubital vein at a flow rate of 3.5 ml/s, followed by a 20 ml saline flush. Scanning of the renal cortex was initiated after a 28-second delay, renal parenchyma imaging after a 65-second delay, and the excretory phase was completed within 3 minutes post-injection. The scan coverage extended from the top of the diaphragm to the iliac crest.

After the CT scan was completed and the obtained images met the diagnostic criteria, the anteroposterior, left-right, and superior-inferior diameters of the space-occupying lesions were measured on the PACS workstation in both transverse and coronal planes. The volume of each lesion was calculated using the formula: volume = (anteroposterior diameter × left-right diameter × superior-inferior diameter) × π/6. The net increment of the CT value for each lesion was determined in both the parenchymal phase (calculated as the difference between the CT values in the renal parenchymal phase and the plain scan phase) and the excretory phase (calculated as the difference between the CT values in the renal excretory phase and the plain scan phase). Each measurement was performed three times, and the average value was recorded (**Figure 1**).

### 2.5 Statistical analysis

Statistical analysis was conducted using SPSS 23.0 software. The normality of the data was assessed using the Kolmogorov-Smirnov (K-S) test. Continuous variables following a normal distribution were expressed as mean ± standard deviation ( $\bar{\text{x}}$ ± SD) and analyzed using the paired *t*-test for comparisons between the two groups. For data that did not follow a normal distribution, results were presented as median (interquartile range) [M (P25, P75)]. Since the continuous variables of interest (mass volume and net increment of CT values) were not normally distributed, Spearman correlation analysis was performed to evaluate the relationships between the GFR change amplitude (%) and mass volume, as well as the net increment of CT values in the parenchymal and excretory phases. A *p*-value of less than 0.05 was considered statistically significant.

## 3 Results

### 3.1 GFR comparison between the diseased kidney and the healthy kidney

The mean GFR of the diseased kidneys (30.84 ± 8.87 mL/min) was significantly lower than that of the contralateral healthy kidneys (38.57 ± 9.48 mL/min), with a mean reduction of 7.73 mL/min (paired *t*-test, *t* = 5.46, *p* < 0.001) (**Table 2**, **Figure 1**).

**Table 2:** Comparison of glomerular filtration rate (GFR) between diseased and healthy kidneys.

Group	Case number (n)	GFR (ml/min)
Healthy kidney	50	38.57 ± 9.48
Diseased kidney	50	30.84 ± 8.87
t		5.46
*p*		<0.01

### 3.2 Correlation analysis

**Figure 2:**
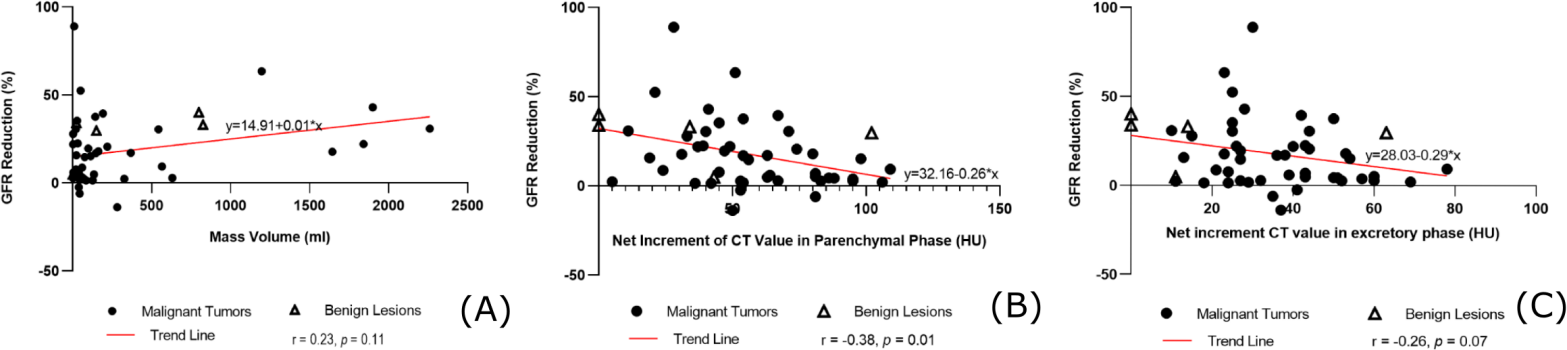
Correlation analysis between CT enhancement characteristics and GFR change. **(A)** Scatter plot showing no significant correlation between renal mass volume and the amplitude of GFR reduction (r = 0.23, *p* = 0.11). **(B)** Scatter plot demonstrating a significant negative correlation between the net increment of CT value in the parenchymal phase and the GFR reduction (Spearman’s r = -0.38, *p* = 0.01). The trend line (red) was generated based on nonlinear fitting of all 50 data points to illustrate the overall relationship. Solid circles represent malignant tumors; triangles represent benign masses. This supports the hypothesis that greater tumor vascularity is associated with a smaller apparent reduction in SPECT-GFR (i.e., a greater overestimation of true function). **(C)** Scatter plot showing no significant correlation between the net increment of CT value in the excretory phase and GFR reduction (r = -0.26, *p* = 0.07).

Spearman correlation analysis revealed no significant association between the volume of the renal masses (median [IQR]: 75.7 cm³ [24.6–282.3]) and the percentage reduction in GFR (r = 0.23, *p* = 0.11). However, a statistically significant moderate negative correlation was observed between the net increment of CT value in the parenchymal phase (median [IQR]: 53 HU [37-80]) and the GFR reduction (r = -0.38, *p* = 0.01). In contrast, the net increment in the excretory phase showed no significant correlation with GFR change (r = -0.26, *p* = 0.07) (**Table 3** & **Figure 2**).

**Table 3:** The correlation of GFR change amplitude with the volume of the mass and net increment of CT value of mass (n=50).

Parameters	r	*p*
Volumes of masses	0.23	0.11
Net increments of CT value of masses in the parenchymal phase	-0.38^**^	0.01
Net increments of CT value of masses in the excretory phase	-0.26	0.07

**denotes statistical significance.

## 4 Discussion

GFR refers to the volume of ultrafiltrate, or primary urine, produced by both kidneys per unit time (per minute), which is a very sensitive indicator of renal function (6). While the measurement of inulin clearance is regarded as the “gold standard” for determining GFR, its clinical application is limited due to inulin’s limited availability, high cost, and the invasive and complex nature of the procedure. As an alternative, estimation equations using endogenous filtration markers, such as serum creatinine have been used in clinical practice; however, these methods may suffer from imprecision and inaccuracies (7, 8). In contrast, SPECT renal dynamic imaging offers a simple, non-invasive, and accurate approach, providing simultaneous information on renal blood perfusion, unilateral renal GFR and renogram, which is often used as an important method for quantitative evaluation of renal function in clinical practice (9–12).

As highlighted in the joint SNMMI/EANM practice guideline, renal scintigraphy is primarily a functional imaging modality and lacks the spatial resolution to provide detailed anatomical information, which is a recognized limitation in its clinical application (13). Moreover, the accuracy of renal dynamic imaging can be influenced by numerous factors, including the delineation of regions of interest and the dosage of the administered tracer (14).

As a routine and preferred preoperative examination for patients with kidney tumors, enhanced multi-slice spiral CT can accurately and efficiently provide detailed information regarding the morphology, density, blood supply, and perirenal anatomy of the diseased kidney (15, 16). In this study, ^99m^Tc-DTPA renal dynamic imaging, combined with enhanced CT scanning, was used to analyze the influence of renal masses on GFR, and to provide a foundation for more accurate renal function evaluation and to guide clinical decision-making.

In all the selected cases, there was no evident compression of the renal sinus or involvement of the renal pelvis. Compression of the renal sinus or renal pelvis by a space-occupying lesion can cause urinary tract obstruction, resulting in hydronephrosis. This condition increases glomerular capsular pressure and reduces glomerular effective filtration pressure, while hydronephrosis-induced compression of the renal parenchyma and thinning of the renal cortex further contribute to a decline in GFR. Studies indicate that GFR measurement lacks accuracy in the patients with severe hydronephrosis but remains reliable in those with mild hydronephrosis (17).

In healthy adults, renal parenchymal volume is strongly correlated with GFR, reflecting its adaptation to metabolic demands and its close association with renal function (18). In patients with renal tumors, moderate correlations have been observed between renal parenchymal volume and GFR both before and after surgery (19–21). The percentage of parenchymal volume preserved (PPVP) is a critical factor influencing postoperative renal function after partial nephrectomy (PN). Larger and more complex tumors are strongly associated with reduced PPVP; however, meticulous surgical precision can mitigate this effect to some degree (22, 23).

A study showed that tumor volume significantly impacts GFR when measured using enhanced CT scanning, as the CT-GFR value—determined by subtracting the CT-GFR measurement without a renal tumor from the CT-GFR measurement with a renal tumor—was strongly correlated with renal tumor volume (24). However, in our study, while the GFR in the diseased kidney was lower than that in the contralateral healthy kidney, no correlation was observed between the size of the space-occupying mass and the decline in GFR in the diseased kidney. This discrepancy may be attributed to the fact that all cases in our study involved localized masses, predominantly small endogenous masses, whereas larger masses caused extrusion of normal renal parenchyma and expansion outside the kidney, primarily resulting in kidney displacement rather than significant parenchymal volume reduction. These conditions led to no notable difference in GFR reduction relative to the volume reduction in renal parenchyma. Therefore, the renal GFR should not be assessed preoperatively solely based on the volume of the renal mass, as this approach may inaccurately influence the determination of the surgical plan.

In SPECT renal dynamic imaging, the renal blood perfusion phase occurs within the first minute after radiopharmaceutical injection, corresponding to the renal parenchymal phase in contrast-enhanced CT. The renal function phase in SPECT begins after the first minute, aligning with the renal parenchymal phase in CT. Delayed SPECT imaging at 3 minutes corresponds to the excretory phase in CT. When renal masses exhibit high enhancement (net increment CT value) in the parenchymal phase of CT, they may also accumulate significant amounts of the ^99m^Tc-DTPA tracer during the SPECT functional phase. Consequently, the radioactive counts originating from the mass itself are inadvertently included in the total renal counts, leading to an overestimation of the kidney’s radioactivity and a consequent underestimation of the true GFR. Thus, the decline in GFR is negatively correlated with the net increments of CT values in the renal masses during the renal parenchymal phase of CT. During delayed SPECT imaging, the radioactive counts within the masses may affect the decline rate of the excretory segment of the renogram but do not affect the calculation of GFR value. Consequently, the decline in GFR is not correlated with the net increments of CT values in the masses during the excretory phase of CT.

While this study focused on the influence of vascularity on dynamic GFR measurement, future investigations could incorporate agents like ^99m^Tc-DMSA, which binds to renal tubules, to separately assess the functional parenchymal volume and further disentangle the contributions of tumor perfusion versus functional renal tissue loss to the observed GFR changes.

When renal tumors are malignant, they secrete vascular growth factors that promote the infiltration of tumor neovascular plexus and the expansion and disorganized growth of the tumor (25, 26). Therefore, renal malignant tumors often show increased blood perfusion, leading to a higher concentration of contrast agent within the tumor during enhanced CT scanning, resulting in an elevated CT value. When a tumor demonstrates high enhancement in the parenchymal phase of CT, it indicates a rich internal vascular supply and delayed clearance of the contrast agent. However, the pathological grade of the tumor can influence not only the degree of enhancement and the rate of contrast agent clearance during CT scanning but also the calculation of the GFR (27–29). In this study, the reduction in GFR in clear cell carcinomas of the same size varied depending on differences in blood supply. Consistent with the proposed mechanism, the apparent GFR reduction was less pronounced (i.e., the GFR was more overestimated) in kidneys with masses exhibiting larger net increments of CT value in the parenchymal phase. In this study, 2 cases of cysts showed no enhancement in any phase of the scan, and the derived GFR reduction in these cases was significantly greater, at 40.1% and 34%, respectively.

Our findings suggest that in patients with highly vascularized renal masses, the GFR measured by SPECT might overestimate the true function of the diseased kidney. Clinicians should be cautious when using this GFR value to plan for partial nephrectomy, as the postoperative renal function might be worse than predicted. Integrating enhancement characteristics from CT can help in making a more accurate functional assessment. Therefore, in clinical practice, a highly enhancing renal mass on CT should alert the clinician that the SPECT-derived GFR for that kidney may be optimistically biased, and this should be factored into surgical planning, particularly when considering nephron-sparing surgery.

Given the relatively small and uneven sample size (e.g., predominance of clear cell carcinoma and right-sided masses), the results of this study should be interpreted as preliminary. This limitation precludes robust subgroup analyses across different pathological subtypes or locations (left *vs*. right kidney). Furthermore, the retrospective design may introduce selection bias. To address these constraints, our research team intends to expand the sample size in future prospective, multi-center investigations, enabling a more robust and comprehensive evaluation of the relationship between GFR changes and mass characteristics. Additionally, we plan to incorporate multiple comparison correction tests in future analyses to strengthen the statistical rigor. A more extensive correlation analysis integrating broader clinical data will also be undertaken to provide a valuable reference for the establishment of predictive renal mass models.

## 5 Conclusion

Renal masses are associated with a reduced GFR in the diseased kidney compared to the healthy kidney. The blood supply to these renal space-occupying masses can be assessed using CT scan. During SPECT renal dynamic imaging, the blood supply of renal masses can affect the calculation of GFR values. Specifically, the abundant blood supply and delayed clearance associated with malignant tumors may result in an overestimation of the GFR in the diseased kidney, making the measured GFR value higher than the actual GFR. Therefore, it is essential to objectively analyze these findings in clinical practice to ensure accurate interpretation and diagnosis.

## Data Availability

The raw data supporting the conclusions of this article will be made available by the authors, without undue reservation.

## References

[1] Lamb EJ Barratt J Brettell EA Cockwell P Dalton RN Deeks JJ . Accuracy of glomerular filtration rate estimation using creatinine and cystatin C for identifying and monitoring moderate chronic kidney disease: the eGFR-C study. Health Technol Assess. (2024) 35:1–169. doi: 10.3310/HYHN1078, PMID: 39056437 PMC11331378

[2] Salvador CL Tøndel C Rowe AD Bjerre A Brun A Brackman D . Estimating glomerular filtration rate in children: evaluation of creatinine- and cystatin C-based equations. Pediatr Nephrol. (2019) 2:301–11. doi: 10.1007/s00467-018-4067-3, PMID: 30171354

[3] Weinert LS Camargo EG Soares AA Silveiro SP . Glomerular filtration rate estimation: performance of serum cystatin C-based prediction equations. Clin Chem Lab Med. (2011) 11:1761–71. doi: 10.1515/CCLM.2011.670, PMID: 21756163

[4] Pang X Li F Huang S Wang C Zhang T Hu Z . A novel method for accurate quantification of split glomerular filtration rate using combination of tc-99m-DTPA renal dynamic imaging and its plasma clearance. Dis Markers. (2021) 2021:6643586. doi: 10.1155/2021/6643586, PMID: 33791044 PMC7984922

[5] Gates GF . Split renal function testing using Tc-99m DTPA. A rapid technique for determining differential glomerular filtration. Clin Nucl Med. (1983) 9:400–7. doi: 10.1097/00003072-198309000-00003, PMID: 6357589

[6] Peters AM . The kinetic basis of glomerular filtration rate measurement and new concepts of indexation to body size. Eur J Nucl Med Mol Imaging. (2004) 1:137–49. doi: 10.1007/s00259-003-1341-8, PMID: 14593500

[7] Carvalho JP Marques A Abreu F Pintão S . Comparison of GFR measurement with a two-blood sample technique using [99mTc]Tc-DTPA vs. creatinine-based equations in potential kidney donors. J Bras Nefrol. (2023) 3:344–9. doi: 10.1590/2175-8239-JBN-2022-0105en, PMID: 36445202 PMC10697154

[8] Miladinova D Makazlieva T Peshevska A Rambabova-Bushljetik I Poposka D Majstorov V . The current state of nuclear nephrology in modern medicine. Pril (Makedon Akad Nauk Umet Odd Med Nauki). (2023) 3:7–16. doi: 10.2478/prilozi-2023-0042, PMID: 38109445

[9] Spiliotopoulou M Papathanasiou N Łabieniec Ł Papachristou E Fokaefs E Spyridonidis T . 99mTc-DTPA dynamic SPECT/CT renogram in adults: feasibility and diagnostic benefit. Nucl Med Commun. (2024) 8:673–82. doi: 10.1097/MNM.0000000000001865, PMID: 38779747

[10] Kang YK Park S Suh MS Byun SS Chae DW Lee WW . Quantitative single-photon emission computed tomography/computed tomography for glomerular filtration rate measurement. Nucl Med Mol Imaging. (2017) 4:338–46. doi: 10.1007/s13139-017-0491-8, PMID: 29242728 PMC5721092

[11] Yuan X Tang W Shi W Yu L Zhang J Yuan Q . Determination of glomerular filtration rate (GFR) from fractional renal accumulation of iodinated contrast material: a convenient and rapid single-kidney CT-GFR technique. Eur Radiol. (2018) 7:2763–71. doi: 10.1007/s00330-017-5289-7, PMID: 29426992

[12] Gibson P Shammas A Cada M Licht C Gupta AA . The role of Tc-99m-DTPA nuclear medicine GFR studies in pediatric solid tumor patients. J Pediatr Hematol Oncol. (2013) 2:108–11. doi: 10.1097/MPH.0b013e31825804b2, PMID: 22767127

[13] Taylor AT Brandon DC De Palma D Blaufox MD Durand E Erbas B . The SNMMI procedure standard/EANM practice guideline for diuretic renal scintigraphy in adults with suspected upper urinary tract obstruction 1.0. Semin Nucl Med. (2018) 4:377–90. doi: 10.1053/j.semnuclmed.2018.02.010, PMID: 29852947 PMC6020824

[14] Assadi M Eftekhari M Hozhabrosadati M Saghari M Ebrahimi A Nabipour I . Comparison of methods for determination of glomerular filtration rate: low and high-dose Tc-99m-DTPA renography, predicted creatinine clearance method, and plasma sample method. Int Urol Nephrol. (2008) 4:1059–65. doi: 10.1007/s11255-008-9446-4, PMID: 18690545

[15] Nguyen KA Brito J Hsiang W Nolte A Syed JS Suarez-Sarmiento A . National trends and economic impact of surgical treatment for benign kidney tumors. Urol Oncol. (2019) 3:183.e9–183.e15. doi: 10.1016/j.urolonc.2018.11.019, PMID: 30587401

[16] Marcon J Graser A Horst D Casuscelli J Spek A Stief CG . Papillary vs clear cell renal cell carcinoma. Differentiation and grading by iodine concentration using DECT-correlation with microvascular density. Eur Radiol. (2020) 1:1–10. doi: 10.1007/s00330-019-06298-2, PMID: 31278580

[17] Yin H Liang W Zhao D . The application value of the renal region of interest corrected by computed tomography in single-kidney glomerular filtration rate for the evaluation of patients with moderate or severe hydronephrosis. Front Physiol. (2022) 13:861895. doi: 10.3389/fphys.2022.861895, PMID: 35615674 PMC9124959

[18] Johnson S Rishi R Andone A Khawandi W Al-Said J Gletsu-Miller N . Determinants and functional significance of renal parenchymal volume in adults. Clin J Am Soc Nephrol. (2011) 1:70–6. doi: 10.2215/CJN.00030110, PMID: 20847095 PMC3022251

[19] Liu W Zhu Y Zhu X Yang G Xu Y Tang L . CT-based renal volume measurements: correlation with renal function in patients with renal tumours. Clin Radiol. (2015) 12:1445–50. doi: 10.1016/j.crad.2015.09.005, PMID: 26454346

[20] Xiong L Zou X Luo X Yin S Huang Y Ning K . Longitudinal changes in renal parenchymal volume and function status after partial nephrectomy: a retrospective cohort study. Int J Surg. (2024) 2:984–91. doi: 10.1097/JS9.0000000000000938, PMID: 38000077 PMC10871623

[21] Funahashi Y Hattori R Yamamoto T Kamihira O Sassa N Gotoh M . Relationship between renal parenchymal volume and single kidney glomerular filtration rate before and after unilateral nephrectomy. Urology. (2011) 6:1404–8. doi: 10.1016/j.urology.2010.03.063, PMID: 20570320

[22] Kazama A Attawettayanon W Munoz-Lopez C Rathi N Lewis K Maina E . Parenchymal volume preservation during partial nephrectomy: improved methodology to assess impact and predictive factors. BJU Int. (2024) 2:219–28. doi: 10.1111/bju.16300, PMID: 38355293

[23] Marconi L Desai MM Ficarra V Porpiglia F Van Poppel H . Renal preservation and partial nephrectomy: patient and surgical factors. Eur Urol Focus. (2016) 6:589–600. doi: 10.1016/j.euf.2017.02.012, PMID: 28723490

[24] Wang T Xu Y Liu W Shao P Lv Q Yang G . Measurement of glomerular filtration rate using multiphasic computed tomography in patients with unilateral renal tumors: A feasibility study. Front Physiol. (2019) 10:1209. doi: 10.3389/fphys.2019.01209, PMID: 31607949 PMC6761270

[25] Hemmerlein B Kugler A Ozisik R Ringert RH Radzun HJ Thelen P . Vascular endothelial growth factor expression, angiogenesis, and necrosis in renal cell carcinomas. Virchows Arch. (2001) 5:645–52. doi: 10.1007/s004280100464, PMID: 11764385

[26] Song KH Song J Jeong GB Kim JM Jung SH Song J . Vascular endothelial growth factor - its relation to neovascularization and their significance as prognostic factors in renal cell carcinoma. Yonsei Med J. (2001) 5:539–46. doi: 10.3349/ymj.2001.42.5.539, PMID: 11675683

[27] Ficarra V Caloggero S Rossanese M Giannarini G Crestani A Ascenti G . Computed tomography features predicting aggressiveness of Malignant parenchymal renal tumors suitable for partial nephrectomy. Minerva Urol Nephrol. (2021) 1:17–31. doi: 10.23736/S2724-6051.20.04073-4, PMID: 33200903

[28] Wang K Dong L Li S Liu Y Niu Y Li G . CT features based preoperative predictors of aggressive pathology for clinical T1 solid renal cell carcinoma and the development of nomogram model. BMC Cancer. (2024) 1:148. doi: 10.1186/s12885-024-11870-1, PMID: 38291357 PMC10826073

[29] Wu K Wu P Yang K Li Z Kong S Yu L . A comprehensive texture feature analysis framework of renal cell carcinoma: pathological, prognostic, and genomic evaluation based on CT images. Eur Radiol. (2022) 4:2255–65. doi: 10.1007/s00330-021-08353-3, PMID: 34800150

